# Author Correction: Self‑organized wavy infection curve of COVID‑19

**DOI:** 10.1038/s41598-021-90066-0

**Published:** 2021-05-18

**Authors:** Takashi Odagaki

**Affiliations:** 1 Kyushu University, Fukuoka, 819‑0395 Japan; 2 Research Institute for Science Education, Inc., Kyoto, 603‑8346 Japan

Correction to: *Scientific Reports* https://doi.org/10.1038/s41598-021-81521-z, published online 21 January 2021

The Acknowledgements section in this Article was omitted. The Acknowledgements section should read:

“This work was supported in part by JSPS KAKENHI Grant Number 18K03573."

